# Pharmacological content in the television series “House MD”: analysis from a German pharmacologist’s perspective

**DOI:** 10.1007/s00210-026-04995-6

**Published:** 2026-02-07

**Authors:** Erika Schmoll, Roland Seifert

**Affiliations:** Institute of Pharmacology, Hannover Medical School, Berlin, Carl-Neuberg-Str. 1, D-30625 Hannover, Germany

**Keywords:** Dr. House, TV series, Pharmacological content, Patient education

## Abstract

Nowadays, the media make a major contribution to the medical education of the population. However, people who are not experts are hardly able to assess the accuracy of the situations depicted. To test the reliability of the statements made, this paper deals with the analysis of the accuracy of the pharmacological content in the television series *Dr. House*. The database is currently around 20 years old. The series was first broadcasted in the United States in 2004. The aim of this study was to check the quality of the pharmacological content. In addition, it analyzed the education by the TV series *Dr. House* in the field of pharmacological knowledge. Ten episodes containing 105 pharmacological items were analyzed. The analysis was illustrated with eleven comparable parameters of the items. This includes, for example, naming the drugs, evaluating the indication, assessing the therapy, and presenting the drug within the scene. In addition, the course of the series was analyzed for the first and eleventh episodes of each season. This resulted in 131 additional pharmacological contents. The study shows a high usage rate of international non-proprietary names (INN). The indications are often correctly stated, and an appropriate therapy is initiated. A particular and quite unusual feature of the series is that drugs are used as diagnostic tools to come to the correct diagnosis. Antibacterial drugs and psychotropic drugs are strongly presented. The analysis also highlights differences in the use of drugs between various genres in which drugs play an important role. Due to the cynical character traits of Dr. House, there were issues of concerns such as lack of patient information and use of dangerous/experimental therapies. There were also several medication errors. The way of drug administration often remained unclear and the mechanism of action was not explained. A comparison of the seasons throughout the series reveals a decline in the quality of naming, explanation, and accuracy of indications and presentation of pharmacological content. The lowest quality is evident in season 6, with a marked improvement in seasons 7 and 8. Despite some concerns regarding ethical issues, the TV series *Dr. House* makes a valuable contribution to introducing to the public a broad diversity of pharmacological topics.

**Supplementary Information:**

The online version contains supplementary material available at 10.1007/s00210-026-04995-6.

## Introduction

*Dr. House* is an US American television series that portrays the eponymous protagonist Dr. Gregory House, head of the Department of Diagnostic Medicine at Princeton-Plainsboro University Hospital. The original title is “House MD”. Dr. House shows idiosyncratic and cynical behavior. The character avoids patient contact, disregards basic hospital rules and is addicted to the medication Vicodin® (hydrocodone + paracetamol). He is characterized by its outstanding diagnostic accuracy. Eight seasons with a cumulative 177 episodes were broadcasted. The first broadcast took place on November 16, 2004 in the United States. Broadcasting on German television began on May 9, 2006 (Wikipedia (Germany), (Wikipedia 2025))*. Dr. House* is usually supported by three other medical colleagues in the diagnostic department. Several patients are presented per episode, with a focus on one main patient. A medical advisory team consisting of four medical doctors supported the scriptwriters in researching the case presentations.

The first season of *Dr. House* was reached around 3.59 million viewers in Germany (Quotenmeter.de 2007). The number of viewers increased steadily. As a result, the TV series *Dr. House* has had a major impact on the possibility of medical education for viewers and provides an insight into everyday medical life at Princeton-Plainsboro University Hospital.

The TV series *Dr. House* was not only very popular with the general public, but also in medical circles. In addition to winning several Emmy Awards and two Golden Globes (Wikipedia (Germany), (Wikipedia 2025)), the series also received positive criticism for its medical accuracy by specialist staff. The professional qualities of *Dr. House* met with praise, while the interpersonal skills seem to serve as a counterexample. Table S1 shows a selection of reviews of the television series Dr. House in various German print media. The assessments marked in yellow show a rather critical attitude of the authors towards the role model behavior of the television series. Assessments marked in green represent positive criticism.

The aim of this study is to examine the correctness and thus the quality of the pharmacological content presented and to demonstrate this with regard to the naming of the active ingredients, their presentation and the dosage form as well as the resulting benefits for patient education. The provision of information prior to therapies and the intended use will also be examined.

The following specific questions were addressed:

1. How are drugs named and presented?
2. To what extent does the presentation correspond to reality?
3. Is the indication for the administration of the drug correct according to the state of knowledge at that time and today?
4. Is sufficient patient information provided?
5. Is the TV series *Dr. House* more realistic compared to other media, such as the TV series *Tatort (crime scene)* and selected crime novels?
6. Has the quality of the pharmacological content changed over the course of the series?

## Material and methods

### Analysis of the individual episodes of Dr. House

This paper analyzes the first ten episodes of the first season of *Dr. House*. In table S2, these episodes are listed with the main diagnosis and first broadcast date. Here, 105 pharmacological contents, hereinafter referred to as items, were considered. The first and eleventh episodes of each season were also reviewed and analyzed considering the selected parameters.

**Fig. 1:**
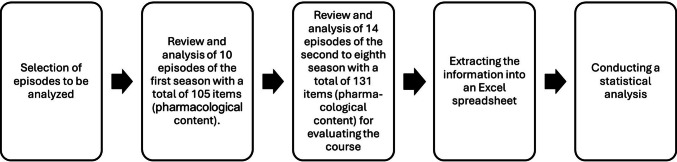
Illustration of the procedure for collecting and analyzing the collected data in a flowchart

Figure 1 shows the analysis procedure using a flow chart. The analysis was illustrated with eleven comparable parameters of the items. The following parameters were taken into account:

1. Naming of the drug
2. Evaluation of the indication
3. Assessment of the therapy
4. Carrying out the reconnaissance
5. Effect on the audience
6. Prescription by the team
7. Classification into drug groups
8. Intended use
9. Representation of the drug within the scene
10. Dosage form of the medicinal product
11. Frequency of diagnosis

This was followed by a fact check with examples of correct content and incorrect scenes. The data was categorized with current findings from the "Encyclopedia of Molecular Pharmacology—Third Edition" (Offermanns and Rosenthal 2021) and the "General and Special Pharmacology and Toxicology" (Aktories et al. 2022). In addition, a comparison was made with information from the time of the first broadcast using the specialist literature "Goodman & Gilman's: The Pharmacological Basis of Therapeutics, eleventh edition" (Brunton et al. 2006) from 2006, as well as current guidelines.

### Comparison with data from the Drug Prescription Report (DPR)

The data from the DPR 2005 (Schwabe and Paffrath 2006), as an indicator for the drug prescription at the time of the broadcast, and the DPR of 2023 (Wissenschaftliches Institut der AOK (WIdO) 2023), served as a current comparison.

### Comparison with the crime scene and crime novels

Subsequently, these findings were compared with the analyses of the German TV series *Tatort* by Ellerbeck and Seifert (Ellerbeck and Seifert 2022) on the topic of poisoning cases and the analysis of pharmacological content by Borchert and Seifert (Borchert and Seifert 2023) as a genre comparison. A further comparison was made by examining the pharmacological content of selected crime novels by Möller and Seifert (Möller and Seifert 2024). The analysis parameters listed above were used in each case.

## Results and discussion

### Naming of the drug

**Fig. 2:**
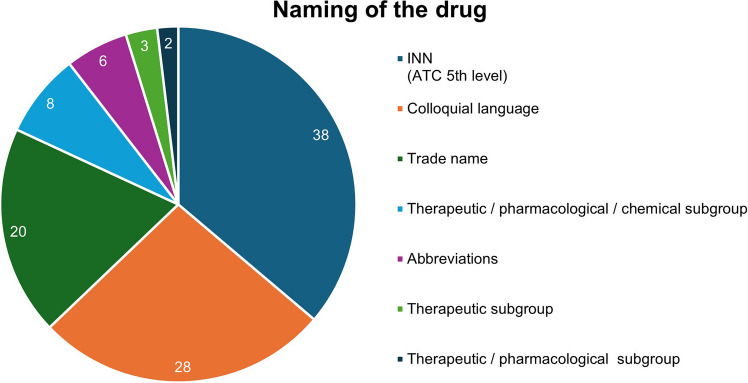
Naming of the drugs according to the ATC code

The distribution of the names of the drug is shown in Fig. 2. It reveals that 38 of the 105 items are presented according to the current WHO recommendation using the active substance name, the so-called INN (International Nonproprietary Name), corresponding to the ATC code of level 5. The second most frequently used term is the colloquial name, followed by 20 items that are represented by trade names. Levels 2, 3 and 4 of the ATC code, which are represented by generic terms in the classification system, are represented to a lesser extent.

The ATC system, which classifies the drugs that can be prescribed in Germany, is shown in table S3. The German classification is carried out by the Scientific Institute of the AOK (WIdO, 2025).

### Assessment of the correctness of the presentation

**Fig. 3:**
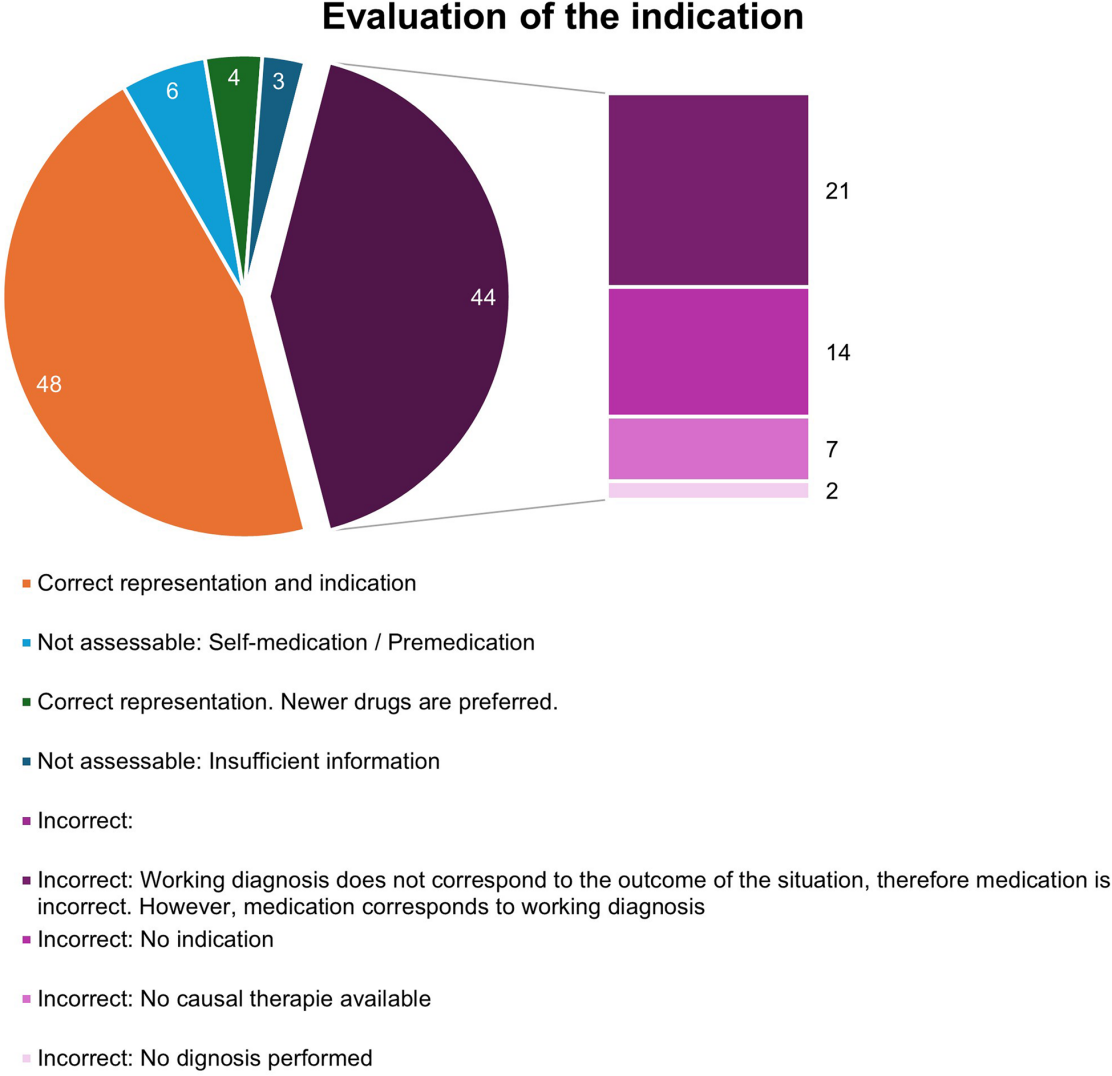
Correctness of indication and presentation of pharmacological content

An important focus of the analysis is to determine the correctness of the presentation of the pharmacological content. This partial analysis is shown graphically in Fig. 3. 48 items are presented correctly and there is an indication for this drug. In a total of 44 pharmacological contents, the presentation is incorrect. In 21 of the latter items, the working diagnosis does not correspond to the outcome of the situation, but the medication is appropriate for the working diagnosis. In the pharmacological content presented, there were only minor deviations in correctness compared to current literature sources at the time the series was broadcast. An overview of the comparison is given in table S4.

Accordingly, 69.5% of the pharmacological contents show a suitable indication for the working diagnosis or final diagnosis. Table 1 shows a plausibility check with examples of correct content. Content with incorrect representations, which are presented by missing indications, fictitious therapy in the absence of causal treatment options or missing diagnostics, account for 21.9% of the items. These scenes are listed as examples in Table 2. 8.6% cannot be conclusively assessed due to a lack of information.

**Table 1:** Representation of exemplary items with correct content

Episode	Category: Evaluation of the indication	Pharmacological content	Scene presentation—correct content	Fact check
Episode 1, Case 1	Correct presentation and indication	Epinephrine ("Epi 05")	She suffers allergic anaphylaxis to the gadolinium injection. Dr. Foreman orders 0.5 mg epinephrine intravenously	“Adrenaline: The most important drug in the acute treatment of anaphylaxis is adrenaline (epinephrine).” (Ring J, 2021) The administration of 0.1 mg epinephrine as an immediate measure in the event of anaphylactic shock, with repeated administration if necessary, is indicated (Aktories et al. 2022, p. 357)
Episode 3, Case 1	Correct presentation and indication	Sodium channel blockers ("anesthetics")	A sodium channel blocker is used to prepare for the bone marrow puncture	"Sodium channel blockers (“Local anesthetics “) reversibly block the generation and transmission of the action potential via nerve fibers and thus prevent the sensation of pain without eliminating consciousness." (Aktories et al. 2022, p. 233)
Episode 5, Case 1	Correct presentation and indication	Epinephrine	The patient reacts to the diphenhydramine with a life-threatening asthma attack. Dr. House administers 0.1 ml epinephrine. GCR agonists (“Glucocorticoids”) are also prescribed during the course of the attack	“Adrenaline: The most important drug in the acute treatment of anaphylaxis is adrenaline (epinephrine).” (Ring J, 2021) "It is crucial for the successful treatment of type I allergies to recognize early symptoms in good time and to establish safe i.v. access. Epinephrine (“EPI”) is injected via this access after clinical effect. Epinephrine (“EPI”) inhibits mediator secretion from mast cells via the β2AR and relaxes the bronchi." (Seifert 2021, p. 72)
Episode 6, Case 1	Correct presentation and indication	Unfractionated heparin (UFH) ("blood thinner")	The on-duty medical doctor explains the therapy to the son and prescribes UFH ("blood thinners") after a symptomatic DVT with pulmonary embolism	"UFH is used for prophylaxis and treatment of venous and arterial thromboembolic diseases." (Aktories et al. 2022, p. 516)
Episode 7, Case 1	Correct presentation and indication	Melarsoprol	The patient is being treated for the cerebral stage of African trypanosomiasis. Melarsoprol is used for this, which is stored in glass cylinders and has to be administered using special intravenous tubes	“The sufficiently CSF-penetrant drugs used for the second stage of the disease are **melarsoprol**, the only drug available until the 1990 s, and NECT, or nifurtimox-eflornithine combination therapy “ (Fairlamb & al., 2018) "Melarsoprol: The substance was introduced in 1948 as the last representative in therapy and is still the only substance that is effective in the cerebral stage of East African sleeping sickness. It is administered intravenously. Its tolerability is poor, so it is only used for the cerebral stage, although it also covers trypanosomes in the hematolymphoid stage. Other toxic reactions include kidney damage with albuminuria and peripheral neuropathy" (Freissmuth et al. 2020, p. 831) " […] the standard treatment of the CNS phase is melarsoprol […]" (Brunton et al. 2006, p. 1051)
Episode 8, Case 3	Correct presentation and indication	Penicillin	Dr. House prescribes penicillin to the patient with syphilis	“Early syphilis should be treated once with benzathine benzylpenicillin 2.4 million IU intramuscularly (gluteal left/right 1.2 million IU each).” (Schöfer and Brockmeyer 2021) "(High-dose) treatment in short infusions for severe or difficult to treat infections caused by the pathogens in the spectrum of activity, e.g. (…) syphilis, especially neurosyphilis" (Aktories et al. 2022, p. 760) "Therapy of syphilis with penicillin G is highly effective." (Brunton et al. 2006, p. 1136)
Episode 9, Case 1	Correct presentation and indication	Unfractionated heparin (UFH) ("blood thinner")	Dr. Foreman explains to the patient that it is a blood clot in the brain and that this can be treated with UFH, (“blood thinner”). When asked, the side effect of pulmonary hemorrhage is mentioned, which is why the patient refuses the therapy. Alternatively, Dr. Foreman suggested an embolectomy, which was subsequently performed	"Unfractionated heparin is indicated for the prophylaxis and treatment of deep vein thrombosis and pulmonary embolism, for the treatment of arterial thrombosis and embolism and as part of anticoagulation in extracorporeal circulation (hemodialysis, heart–lung machine)." (Freissmuth et al. 2020, p. 485)

**Table 2:** Display of items with incorrect content

Episode	Category: Evaluation of the indication	Pharmacological content	Scene display—Incorrect content	Fact check
Episode 1, Case 1	Working diagnosis does not correspond to the outcome of the situation, therefore incorrect medication. However, medication corresponds to the working diagnosis	Prednisone	Dr. House prescribes high doses of prednisone as he suspects cerebral vasculitis. Unexpected drug effects are accepted by Dr. House as "educational". No explanation is given, only that this is "part of the treatment". When asked several times by the patient, the assumption of vasculitis and treatment with GCR agonists (“steroids”) is explained	"GCR agonists (“Glucocorticoids”) are used widely in the treatment of a variety of rheumatic disorders and are a mainstay in the treatment of the more serious inflammatory rheumatic diseases, such as systemic lupus erythematousus, and a variety of vasculitic disorders, such as polyarteritis nodosa, granulomatosis with polyangiitis (“Wegener's granulomatosis”), eosinophilic granulomatosis with polyangiitis (“Churg-Strauss syndrome”), and giant cell arteritis." (Brunton et al. 2006, p. 1607)
Episode 2, Case 1	Working diagnosis does not correspond to the outcome of the situation, therefore incorrect medication. However, medication corresponds to the working diagnosis	Penicillin	Dr. Cameron suspects neurosyphilis and advises penicillin, which according to Dr. House should be administered intrathecally in order to have a local effect. Dr. Foreman argues that intrathecally administered substances increase intracranial pressure, which can lead to damage. It was neglected here that the patient had an Ommaya reservoir inserted, which equalizes the pressure	“Symptomatic and asymptomatic neurosyphilis should be treated with penicillin G in crystalloid solution at a daily dose of 4 × 6 million, 5 × 5 million, or3 × 10 million IU intravenously for 14 days (at least 10 days).“ (Schöfer and Brockmeyer 2021) (Schöfer and Brockmeyer 2021) "Therapy of syphilis with penicillin G is highly effective." (Brunton et al. 2006, p. 1163)
Episode 3, Case 1	Working diagnosis does not correspond to the outcome of the situation, therefore incorrect medication. However, medication corresponds to the working diagnosis	Antibacterial Chemotherapeutics ("broad-spectrum antibiotics")	A broad-spectrum antibacterial drug (“broad-spectrum antibiotic”) is prescribed for the treatment of sepsis	“We recommend that patients with sepsis or septic shock receive initial anti-infective therapy with one or more antibacterial chemotherapy to cover all major bacteria Note: Anti-infective therapy should be based on the suspected focus of infection, MRE colonization, and medical history.” (DSG (Deutsche Sepsis-Gesellschaft), (DSG 2025)) "The combination of β-lactam antibacterial drug (“antibiotics”) with aminoglycosides has proven to be particularly effective for - (…) - severe sepsis" (Aktories et al. 2022, p. 751)
Episode 3, Case 1	Working diagnosis does not correspond to the outcome of the situation, therefore incorrect medication. However, medication corresponds to the working diagnosis	Unasyn® (Sultamicillin) Levothyroxine	Dr. House suspects a simultaneous occurrence of sinusitis and hypothyroidism, as both diagnoses together cover all symptoms. He prescribes Unasyn and levothyroxine. Unasyn® (Sultamicillin) is described as a targeted antibacterial for sinusitis	Sultamicillin: "Ampicilin + β-lactamase inhibitor is listed as the drug of choice for acute rhinosinusitis." (Aktories et al. 2022, p. 753) "Thyroxine (levothyroxine sodium) is the hormone of choice for thyroid hormone replacement therapy because it is consistent potency and prolonged duration of action. (Brunton et al. 2006, p. 1524)
Episode 4, Case 1	Working diagnosis does not correspond to the outcome of the situation, therefore incorrect medication. However, medication corresponds to the working diagnosis	Aciclovir Ribavirin Antibacterial Chemotherapeutics ("broad-spectrum antibiotics")	Differential diagnoses are discussed in the team. Dr. Foreman notes that the children do not respond to aciclovir and ribavirin. Dr. House also notes that they do not respond to broad-spectrum antibacterial drugs (“broad-spectrum antibiotic”)	"These drugs (aciclovir and valaciclovir) are particularly useful in immunocompromised patients because these individuals experience both more frequent and more severe HSV and VZV infections." "Ribavirin aerosol is approved in the United States for treatment of RSV bronchiolitis and pneumoniae in hospitalized children. […] Intravenous and/or aerosol ribavirin has been used occasionally in treating severe influenza virus infection and in the treatment of immunosuppressed patients with adenovirus, vaccinia, parainfluenza, or measlesvirus infections." "Carbapenems are beta-lactams that contain a fused beta-lactam ring and five membered ring system that differs from the penicillins in being unsaturated and containing a carbon atom instead of the sulfur atom. This class of antibiotics has a broader spectrum of activity than do most other beta-lactam antibacterial Chemotherapeutics (“antibiotics”)." (Brunton et al. 2006, pp. 1249, 1266, 1150)
Episode 4, Case 1	Working diagnosis does not correspond to the outcome of the situation, therefore incorrect medication. However, medication corresponds to the working diagnosis	Vancomycin Aztreonam	Dr. House orders vancomycin for MRSA and aztreonam for other bacterial infections, especially Pseudomonas, Haemophilus influenzae and VRE In the course of the disease, kidney failure occurs without crystal formation, caused by the previously administered antibacterial drugs. As aztreonam and vancomycin can trigger kidney failure, Dr. House decides to discontinue aztreonam in one child and vancomycin in the other. This is done to protect the other affected children	“Vancomycin remains an important standard of care for MRSA infections but is limited with respect to nephrotoxicity and rapid target attainment” (Chang J, 2023) "Vancomycin should be employed only to treat serious infections and is particularly useful in the management of infections due to it methicillin-resistant staphylococci […]." "Aztreonam has activity only against gram-negative bacteria; it has no activity against gram-positive bacteria and anaerobic organisms. However, activity against Enterobacteriaceae is excellent, as is that against P. aeruginosa. It is also highly active in vitro against H. influenzae and gonococci." (Brunton et al. 2006, pp. 1196, 1151)
Episode 5, Case 1	Working diagnosis does not correspond to the outcome of the situation, therefore incorrect medication. However, medication corresponds to the working diagnosis	Prednisone	Dr. House suspects eosinophilic granulomatosis with polyangiitis (“Churg-Strauss vasculitis”) and prescribes prednisone 40 mg 3 times a day	“Its conventional treatment relies mainly on agents that decrease inflammation: corticosteroids and immunosuppressant adjunction for severe manifestations.“ (Raffray L, 2018) "GCR agonists (“Glucocorticoids”) are used widely in the treatment of a variety of rheumatic disorders and are a mainstay in the treatment of the more serious inflammatory rheumatic diseases, such as systemic lupus erythematousus, and a variety of vasculitic disorders, such as polyarteritis nodosa, granulomatosis with polyangiitis (“Wegener's granulomatosis”), eosinophilic granulomatosis with polyangiitis (“Churg-Strauss syndrome”), and giant cell arteritis." (Brunton et al. 2006, p. 1607)
Episode 5, Case 1	Working diagnosis does not correspond to the outcome of the situation, therefore incorrect medication. However, medication corresponds to the working diagnosis	COX inhibitors ("non-steroidal anti-inflammatory drugs")	Dr. Cuddy takes over the case and orders 40% oxygen for the suspected pneumonitis from the hyperbaric treatment (symptom: labored breathing) and COX inhibitors (“non-steroidal anti-inflammatories”) for the rash and joint pain	“The analgesic effects of COX inhibitors are dose dependent and differences in effect are most likely related to wrong dosing of the comparator. For acute pain, the combination […] with paracetamol enhances the analgesic effect without increasing the risk of side effects.“ (Stiller and Hjemdahl 2022) COX inhibitors have a pain-relieving, antipyretic and anti-inflammatory effect (Aktories et al. 2022, p. 209)
Episode 8, Case 1	Working diagnosis does not correspond to the outcome of the situation, therefore incorrect medication. However, medication corresponds to the working diagnosis	Adsorbents ("charcoal tablets") Naloxone	A patient presents with sudden onset of nausea and disorientation. There is bradycardia, a heart rate of 48/min. Foreman reports that the patient is not responding to atropine Dr. House suspects a drug intoxication and orders charcoal tablets and naloxone	"Only if ingestion of potentially toxic quantities is suspected and the foreign substance also binds to charcoal should activated charcoal be given" “Naloxone is a pure, competitive opioid antagonist with the highest affinity for the μ-opioid receptor, allowing for the reversal of opioid effects. Evidence indicates that naloxone antagonizes opioid effects by competitively binding to μ, κ, and σ-opioid receptors in the central nervous system (CNS), displaying the highest affinity for the μ receptor. At lower dosages, naloxone causes minimal blockade of δ and κ-opioid receptors while maintaining a significant blockade of μ-opioid receptors. “ (Jordan MR, 2025) "Naloxone can be given for respiratory depression. Naloxone is an opioid receptor antagonist that reverses all the effects of morphine and can even trigger an acute withdrawal syndrome." (Aktories et al. 2022, pp. 971, 975)
Episode 8, Case 1	Working diagnosis does not correspond to the outcome of the situation, therefore incorrect medication. However, medication corresponds to the working diagnosis	Pralidoxime	The patient is given pralidoxime for pesticide intoxication (organophosphate). Bradycardia occurs, which is treated with electrocardioversion	“Pralidoxime is a medication used in the management and treatment of organophosphate poisoning “ (Gupta R, 2025) "Pralidoxime chloride is the only AChE reactivator currently available in the United States and can be obtained in a parenteral formulation." (Brunton et al. 2006, p. 211)
Episode 9, Case 1	Working diagnosis does not correspond to the outcome of the situation, therefore incorrect medication. However, medication corresponds to the working diagnosis	Antibacterial Chemotherapeutics ("broad-spectrum antibiotics")	The patient is admitted with suspected lobar pneumonia following dyspnea while he had been playing trumpet. Dr. Foreman prescribes broad-spectrum antibacterial drugs (“broad-spectrum antibiotic”)	"Before starting a calculated therapy for community-acquired pneumonia (…), material must be obtained for microbiological diagnostics (…). Since β-lactam antibacterial drugs (“antiobiotics”) are not effective against most pathogens (…), tertacyclines (doxycycline), markolid-antibacterial drugs (“antiobiotics”) or moxifloxacin are indicated." (Aktories et al. 2022, p. 752)
Episode 9, Case 1	Working diagnosis does not correspond to the outcome of the situation, therefore incorrect medication. However, medication corresponds to the working diagnosis	Cytoxan® (cyclophosophamide)	The lung biopsy results show inflammation. Dr. House prescribes and administers Cytoxan® (cyclophosophamide) in cases of Granulomatosis with polyangiitis (“Wegener's disease”)	“In this comparativeness effectiveness study using clinical data, rituximab induction therapy for GPA was more frequently associated with remission than cyclophosphamide. “ (Puéchal X and St, 2022) "It has activity in nonneoplastic disorders associated with altered immune reactivity, including granulomatosis with polyangiitis (“Wegners' granulomatosis”), rheumatoid arthritis, and the nephrotic syndrome." (Brunton et al. 2006, p. 1328)
Episode 10, Case 1	Working diagnosis does not correspond to the outcome of the situation, therefore incorrect medication. However, medication corresponds to the working diagnosis	Isoniazid ("INH") Rifampicin Streptomycin	Dr. House suspects a tuberculoma in the ovarian mass and orders INH, rifampicin and streptomycin without further diagnostics, as there is no treatment for this patient with the differential diagnosis of advanced ovarian cancer	“The treatment of drug-sensitive tuberculosis should consist of eight weeks of isoniazid (INH), rifampicin (RMP), pyrazinamide (PZA), and ethambutol (EMB), followed by four months of isoniazid (INH) and rifampicin (RMP)(total treatment duration six months) Streptomycin is no longer considered part of standard therapy.” (Tom Schaberg 2022) "Rifampicin and isoniazid are the most effective drugs available for the treatment of tuberculosis." "Since other effetive drugs have become available, the use of streptomycin for the treatment of pulmonary tuberculosis has been sharply reduced. Many clinicians prefer to give 4 drugs, of which streptomycin may be one, for the most serious forms of tuberculosis, such as disseminated disease or meningitis." (Brunton et al. 2006, pp. 1208, 1211)
Episode 10, Case 1	Working diagnosis does not correspond to the outcome of the situation, therefore incorrect medication. However, medication corresponds to the working diagnosis	Ceftriaxone	The laboratory results indicate meningitis. Dr. House recommends transfer to an isolation ward and the administration of ceftriaxone	“The initial calculated antibiotic therapy for community-acquired bacterial meningitis in adults involves a combination of ampicillin and a cephalosporin from group 3a (e.g., ceftriaxone).“ (Pfister H.-W. 2023) Ceftriaxone: "Administration of the drug once or twice daily has been effective for patients with meningitis." (Brunton et al. 2006, p. 1148)
Episode 1, Case 4	No diagnostics performed	Placebo	The patient reports headaches and occasional fever. He has looked this up on the internet. He came across chronic fatigue syndrome or fibromyalgia Without any further diagnostics, Dr. House swaps Vicodin® (hydrocodone + paracetamol) pills from the medication box for sugar balls from the gumball machine and uses them as a placebo The patient who has received sugar balls from Dr. House shows up again to receive more pills. It can be assumed that the placebo has helped the patient subjectively	A lack of diagnosis can result in a serious illness being overlooked. In particular, the administration of a placebo without further information should be discussed ethically
Episode 3, Outpatient clinic	No diagnostics performed	Motrin® (Ibuprofen)	Dr. House explains to the patients waiting in the emergency room that he has no desire to treat them, but that “a monkey with a bottle of Motrin® (ibuprofen) can do it.”	A lack of anamnesis, including diagnostics, can result in a serious illness being overlooked. Therefore, a general prescription of ibuprofen is not recommended, especially when considering the ADR profile
Episode 1, Case 1	No indication	Vicodin® (hydrocodone + paracetamol)	Dr. House takes 1 tablet of Vicodin® (hydrocodone + paracetamol). This is not stated, but it is clear from the story that Dr. House is addicted to Vicodin® (hydrocodone + paracetamol)	"In general, clinical experience shows that abstinence from medication is particularly recommended when somatic illnesses are caused by the pathological use of medication." (German Society for Psychiatry and Psychotherapy, Psychosomatics and Neurology e.V. (DGPPN), 2020, p. 232)
Episode 1, Case 2	No indication	MOR agonists ("Pain killer")	The patient reports back pain from a harmless trauma. Dr. House offers him one of his “painkillers”. In this case Vicodin® (hydrocodone + paracetamol)	"Opioids do not seem to expedite return to work in injured workers or improve functional outcomes of acute back pain in primary care.” (Deyo RA, 2015) “[…] MOR agonists (“analgesics”) always should be dosed in a continuous or "around the clock" fashion rather than on an as-needed basis for chronic severe pain" (Brunton et al. 2006, p. 581)
Episode 2, Case 2	No indication	Vicodin® (hydrocodone + paracetamol)	The patient has a superficial inflammation in his leg. Dr. House offers him a Vicodin® (hydrocodone + paracetamol) tablet for the pain. The name is not mentioned, but it is clear from the story	"[…] MOR agonists (“analgesics”) always should be dosed in a continuous or "around the clock" fashion rather than on an as-needed basis for chronic severe pain" (Brunton et al. 2006, p. 581)
Episode 2, Case 1	No indication	Ativan® (Lorazepam)	The patient has auditory hallucinations during intrathecal administration of penicillin. 2 mg Ativan® (lorazepam) i.v. is administered	"Benzodiazepines such as alprazolam are used as tranquilizers to relieve anxiety states, e.g., in generalized anxiety disorder and panic attacks The anxiolytic effects are observed at low doses, suggesting that only a small number of GABAA receptors need to be modulated to obtain the anxiolytic effect." (Offermanns and Rosenthal 2021, p. 315)
Episode 5, Case 1	No indication	Ativan® (Lorazepam)	Sister Augustin suffers from olfactory paresthesia and religious visions, whereupon Dr. Foreman Ativan® (lorazepam) orders	"Benzodiazepines such as alprazolam are used as tranquilizers to relieve anxiety states, e.g., in generalized anxiety disorder and panic attacks The anxiolytic effects are observed at low doses, suggesting that only a small number of GABAA receptors need to be modulated to obtain the anxiolytic effect." (Offermanns and Rosenthal 2021, p. 315)
Episode 6, Case 1	No indication	Haldol® (haloperidol)	Dr. House orders the immediate (sudden) discontinuation of the "psychotropic drugs" to see what psychological complaints are actually present Dr. Foreman then draws blood from the patient. She is uncooperative, which is why Dr. Foreman decides to administer 5 mg Haldol® (haloperidol) intravenously. The patient immediately appears sedated and paralyzed. A blood sample is successfully taken The patient vomits blood. Dr. Foreman states that side effects, especially the bleeding, are not to be expected from the haloperidol used previously and that he only used this as a "chemical sedative"	Haloperidol does not have a primarily sedative effect, as it has little affinity for the H_1_ receptor. High affinities to D_2_, D_3_ and D_4_ receptors are described, resulting in an antipsychotic effect (Freissmuth et al. 2020, pp. 325, 331) (Brunton et al. 2006, p. 464)
Episode 7, Case 1	No indication	Ativan® (Lorazepam)	The patient suffers a seizure. Dr. Cameron immediately orders Ativan® (lorazepam)	“In the case of a single epileptic seizure, a benzodiazepine or a classic seizure suppressant should not be administered either during or after the seizure.” (Holtkamp M*, 2023) "The most prominent of these effects are sedation, hypnosis, decreased anxiety, muscle relaxation, anterograde amnesia, and anticonvulsant activity." (Brunton et al. 2006, p. 402)
Episode 7, Case 1	No indication	Haldol® (Haloperidol)	The patient suffers a panic attack and visual hallucinations, whereupon Dr. Carmeron immediately prescribes Haldol® (aloperidol) 5 mg i.v	"To achieve control of symptoms in the treatment of acute psychoses, the dose of antipsychotic drug is increased as tolerated during the first few days. Parenteral short-acting medication sometimes is indicated for acute agitated patients; 5 mg of haloperidol or fluphenazine, or a comparable dose of another drug, is given intermuscularly." (Brunton et al. 2006, p. 483) Intravenous administration is not recommended
Episode 8, Case 1	No indication	Diazepam	The patient begins to convulse. Dr. Chase immediately orders 10 mg of diazepam	“In the case of a single epileptic seizure, a benzodiazepine or a classic seizure suppressant should not be administered either during or after the seizure.” (Holtkamp M*, 2023) The administration of benzodiazepines is indicated to interrupt a prolonged convulsion, in the sense of status epilepticus. In this case, an uncomplicated seizure can be assumed, which is why the administration of diazepam is not necessary (Offermanns and Rosenthal 2021, p. 146)
Episode 8, Case 2	No indication	Diazepam	Another patient is admitted with identical symptoms (unconsciousness, bradycardia and low oxygen saturation). This patient is in a critical condition. Dr. Chase orders saline, atropine and diazepam	“In the case of a single epileptic seizure, a benzodiazepine or a classic seizure suppressant should not be administered either during or after the seizure.” (Holtkamp M*, 2023) The administration of benzodiazepines is indicated to interrupt a prolonged seizure in the sense of status epilepticus. Preventive administration of diazepam is not indicated (Offermanns and Rosenthal 2021, p. 146)
Episode 10, Case 1	No indication	Ativan® (Lorazepam)	During the neurological examination, the patient convulses. Dr. Wilson immediately orders Ativan® (lorazepam). A low blood glucose level of 38 mg/dl is noticed. D-50 i.v. is administered Dr. Foreman also suspects self-induced hypoglycemia caused by a high dose of insulin In the course of the case, the patient states that she deliberately injected too much insulin so that she could get a place to sleep in a hospital	“In the case of a single epileptic seizure, a benzodiazepine or a classic seizure suppressant should not be administered either during or after the seizure.” (Holtkamp M*, 2023) Hypoglycemia must be ruled out in the event of a seizure. The treatment of choice for hypoglycemia is glucose replacement and not the administration of benzodiazepines. "Severe hypoglycemia can lead to convulsions and coma. […] When hypoglycemia is severe, it should be treated with intravenous glucose or an injection of glucagon." (Brunton et al. 2006, p. 1631)
Episode 10, Case 1	No indication	Bromocriptine	The patient has a fever of 40.6 °C Dr. Cameran suspects a serotonin-syndrome due to the Prozac given. The Prozac is then discontinued and replaced with bromocriptine	"Bromocriptine is a dopamine D2 receptor agonist that is well established as a treatment for Parkinson's disease and pituitary tumors." (Offermanns and Rosenthal 2021, p. 726)
Episode 10, Case 1	No indication	Haldol® (Haloperidol)	The patient cannot be found in her room. However, she was recently given 10 mg of Haldol® (haloperidol) by Dr. Foreman for sedation	Haloperidol does not have a primarily sedative effect, as it has little affinity for the H1 receptor. It has a high affinity for D2, D3 and D4 receptors, resulting in an antipsychotic effect (Freissmuth et al. 2020, pp. 325, 331), (Brunton et al. 2006, p. 464)
Episode 10, Case 1	No indication	Ativan® (Lorazepam)	The patient develops a psychosis. Dr. Foreman prescribes 1 mg Ativan® (lorazepam). Dr. Foreman is bitten by the patient. He has himself vaccinated post-exposure against tetanus. Another psychosis follows. Dr. Foreman injects an unnamed substance, but it can be assumed that it is Ativan® (lorazepam) again	"Benzodiazepines such as alprazolam are used as tranquilizers to relieve anxiety states, e.g., in generalized anxiety disorder and panic attacks The anxiolytic effects are observed at low doses, suggesting that only a small number of GABAA receptors need to be modulated to obtain the anxiolytic effect." (Offermanns and Rosenthal 2021, p. 315)
Episode 2, Case 1	No causal therapy available	Intraventricular Interferon	According to Dr. Foreman, intraventricular interferon can be given to treat SSPE (stage 1, initially stage 2). Due to the previous therapy, a correct diagnosis cannot be made by antibody detection, except by retinal biopsy. Using the Ommaya reservoir, the interferon can be administered intraventricularly	“Subacute sclerosing panencephalitis (SSPE) is a neurodegenerative disorder because of the persistence of mutated measles virus in the central nervous system. Till date, no curative therapy has been established for SSPE “ (Garg and Sharma, Disease-Modifying Therapy in Subacute Sclerosing Panencephalitis: An Area of Darkness, 2023) "Till date, no curative therapy has been established for SSPE" "Although some evidence supports the use of a combination of intraventricular/intrathecal interferon with isoprinosine, this strategy remains out of reach of most patients in our region." (Garg and Sharma 2023)
Episode 3, Case 1	No causal therapy available	Antidote ("FAB fragments")	To treat colchicine poisoning, the patient is given FAB fragments (antigen-binding fragment of an antibody) and Tylenol® (acetaminophen/paracetamol) for the hair that has been pulled out	“Although many researchers in the world performed lots of research, there are currently no specific antidotes for colchicine poisoning. Meanwhile, there are no management guidelines to treat patients with acute colchicine poisoning until now. “ (Wu J, 2022) "There is no specific therapy for acute colchicine poisoning. Supportive measures should be used, particularly fluid repletion." (Brunton et al. 2006, p. 708)
Episode 4, Case 1	No causal therapy available	antiviral drug	The patient is infected with echovirus type 11, using an antiviral drug that has only been tested under laboratory conditions	"There are no specific therapeutic measures available. Therefore, symptomatic treatment is still the main focus. The non-approved antiviral Pleconaril is sometimes used successfully." (Diedrich 2006) "Treatment of enteroviral infection to date has been supportive and focused on preventing and managing complications. More specific therapy has been limited to use of IvIG, which is thought possibly to contain neutralizing antibodies against the child's infecting drug; maternal serum has been used for the same reason. Overall, both have had limited success Pleconaril is particularly active against echovirus 11, the most common drug of enteroviral sepsis syndrome." (Aradottir et al. 2001)
Episode 9, Case 1	No causal therapy available	GCR agonists ("i.v. steroids") Synthroid® (levothyroxine)	According to the patient information, the patient suffers from amyotrophic lateral sclerosis (ALS) and receives intravenous GCR agonists (“steroids”) and Synthroid®	“Amyotrophic lateral sclerosis (ALS) is a rapidly progressive neurodegenerative disorder involving loss of upper and lower motor neurons, with most cases ending in death within 3–5 years of onset. Several molecular and cellular pathways have been identified to cause ALS; however, treatments to stop or reverse disease progression are yet to be found. “ (Johnson SA, 2022) "ALS is not curable—however, various individual treatment options have to be considered for improving survival, symptom control and social participation." (Meyer 2021)
Episode 9, Case 1	No causal therapy available	Immunoglobulins ("IvIG")	Dr. House recommends IvIG for the treatment of pneumonia. The breathing of the patient with ALS deteriorates. This is characterized by rattling breathing noises Dr. Foreman prescribes intravenous heparin and suspects that his IvIG administration has triggered a pulmonary artery embolism	"In recent years, indications for the use of intravenous immuniglobulin (IvIG) have expanded beyond replacement therapy for agammaglobulinemia and other iummunodeficiencies to include a varity of bacterial and viral infections, and an array of autoimmune and inflammatory diseases as diverse as thrombocytopenic purpura, Kawasaki's diseases." (Brunton et al. 2006, p. 1424)
Episode 3, Case 1	Self-medication/pre-medication without indication	Colchicine	Dr. House suspects an intake of colchicine, which explains the symptoms. It is likely that the tablets have been mixed up. This is resolved at the end of the episode Dr. House continues to try to prove the theory of colchicine poisoning and describes the staggered cell decay. He suspects that the drugs (e.g. ecstasy) taken have been diluted with colchicine	“The treatment of an acute gout attack should be carried out promptly with colchicine, […] as the first-line treatment.” ((DGRh), 2024) "The main indication for colchicine is in the prevention of recurrent gout, particularly in the early stages of antihyperuricemic therapy." (Brunton et al. 2006, p. 708) This is a case of drug confusion and thus an accidental intake of colchicine without indication
Episode 7, Case 2	Self-medication/pre-medication without indication	β_2_-adrenoceptor antagonists ("beta blockers")	Dr. House suspects that her husband regularly mixes β_2_-adrenoceptor antagonists ("beta blockers") into the short-breathing patient's food. He suspects that the reason is a reduced libido and wants to achieve the same in the short of breath patient	β2-adrenoceptor antagonists reduce tissue perfusion, which can lead to cold extremities and erectile dysfunction. The exact pathophysiological mechanisms have not yet been clearly identified (Corradetti, et al. 2022) In this case, it is a case of intoxication deliberately induced by a third party
Episode 10, Case 1	Self-medication/pre-medication without indication	Insulin	Dr. Foreman also suspects self-induced hypoglycemia caused by a high dose of insulin The patient states that she deliberately injected too much insulin so that she would have a place to sleep in a hospital	"Insulin is the mainstay for treatment of virtually all type 1 DM and many type 2 DM patients." (Brunton et al. 2006, p. 1625) In this case, the patient is not known to have an insulin-dependent disease. Insulin was administered with the intention of causing self-induced hypoglycemia

### Missing indication and incorrect use

In addition to the development from the working diagnosis to the final diagnosis with incorrect content, cases are presented that neither correspond to the current nor the former guidelines. Most of the incorrect content is due to a missing indication for the drug in the actual final diagnosis. There are 10 items with psychotropic drugs that are used for immediate sedation and treatment of acute visual and auditory hallucinations. One drug frequently used in the presentation is lorazepam. Haloperidol is also used several times for immediate sedation in the episodes analyzed. In a further 7 cases, it is a fictitious therapy in the absence of a causal treatment option. An example of this is a scene in which stage 2 SSPE (subacute sclerosing panencephalitis) is cured using interferon.

In 4 of the cases described, there is inadequate use of a drug. No electrocardiographic monitoring was performed to prevent QTc prolongation during treatment with haloperidol. In the cases that presented a dosage form of haloperidol, it was administered intravenously. This method of administration is currently not recommended (Ärzteblatt 2010).

#### Self-medication without indication

Self-medication without a suitable indication is presented in 3 cases. This includes accidentally ingested drugs as well as intentionally added medication. There is a scene in which a homeless patient deliberately injects herself with a large, unknown amount of insulin to get a place to sleep in a hospital. The patient, who does not have diabetes mellitus, thus caused a self-induced hypoglycemia without an existing indication for insulin. Another scene is the confusion of a “cough suppressant” with colchicine for the treatment of gout in the pharmacy. Figure S1 shows the proportion of pharmacological items prescribed by Dr. House and his team. 28 of the 105 items are represented by self-medication or medication from previous therapy.

#### Lack of diagnostics

Two items are classified as incorrect content due to missing diagnostics. Dr. House assumes that all patients in the emergency department can be treated with Motrin® (ibuprofen) without further diagnostic steps.

### Assessment of the therapy

**Fig. 4:**
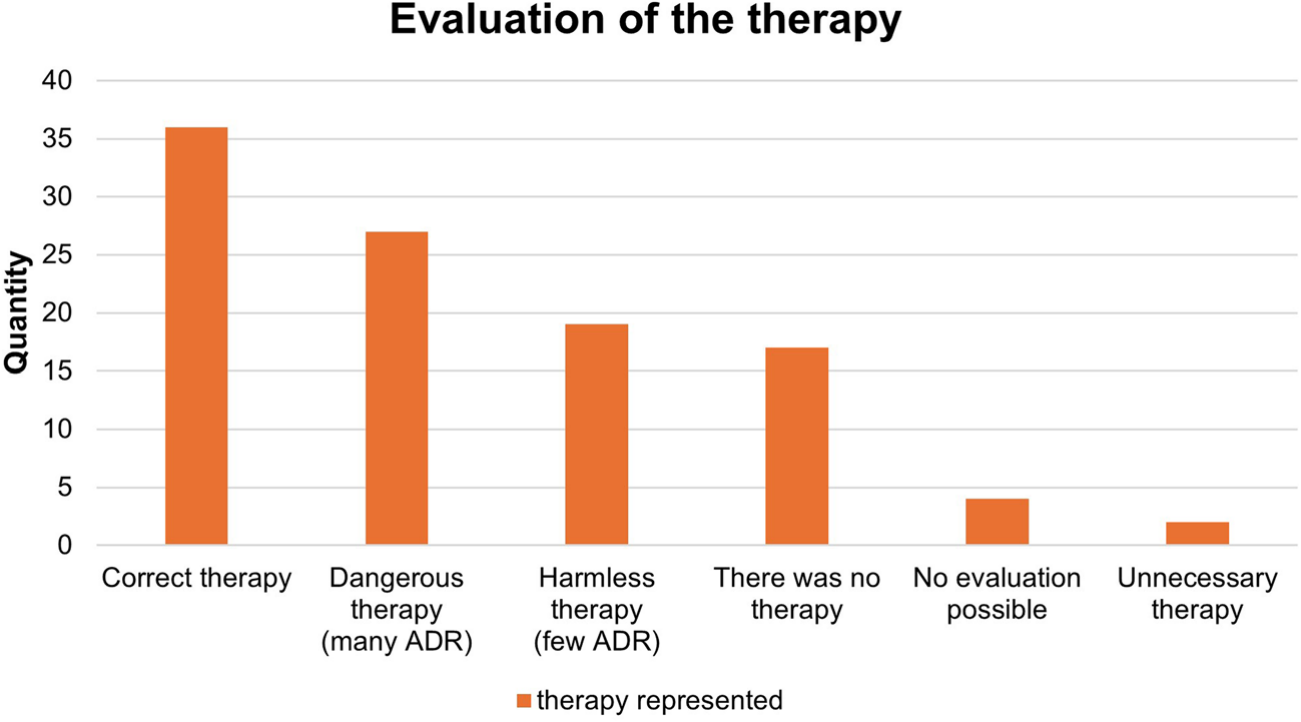
Illustration of the evaluation of the therapies

Figure 4 shows the assessment of the therapy carried out. A correct therapy was applied 36 times. This is reflected as standard therapy in the current literature. In 27 cases, an incorrect therapy, which is to be classified as a dangerous therapy and is associated with the acceptance of many life-threatening or severely damaging adverse drug reactions (ADR). In 17 cases, no therapy was given. These items represent the mention of a drug in the differential diagnostic discussion in the team. Pharmacological treatments with a low ADR profile are classified as harmless therapy. In this case, ADR are to be expected, which are associated with a short-term deterioration or discomfort with spontaneous recovery. This was the case for 19 items. 2 items represent unnecessary treatment. In another 4 cases, no assessment could be made.

In the analysis of the effect on the audience, 58 items are realistic. 18 items trivialize dangerous therapies. Likewise, 18 pharmacological items trivialize the situation by failing to make a diagnosis. Only one scene shows an excessive dramatization of illnesses, with eight scenes presenting a dramatization of therapies. The graphical representation is shown in figure S2.

### Patient information

**Fig. 5:**
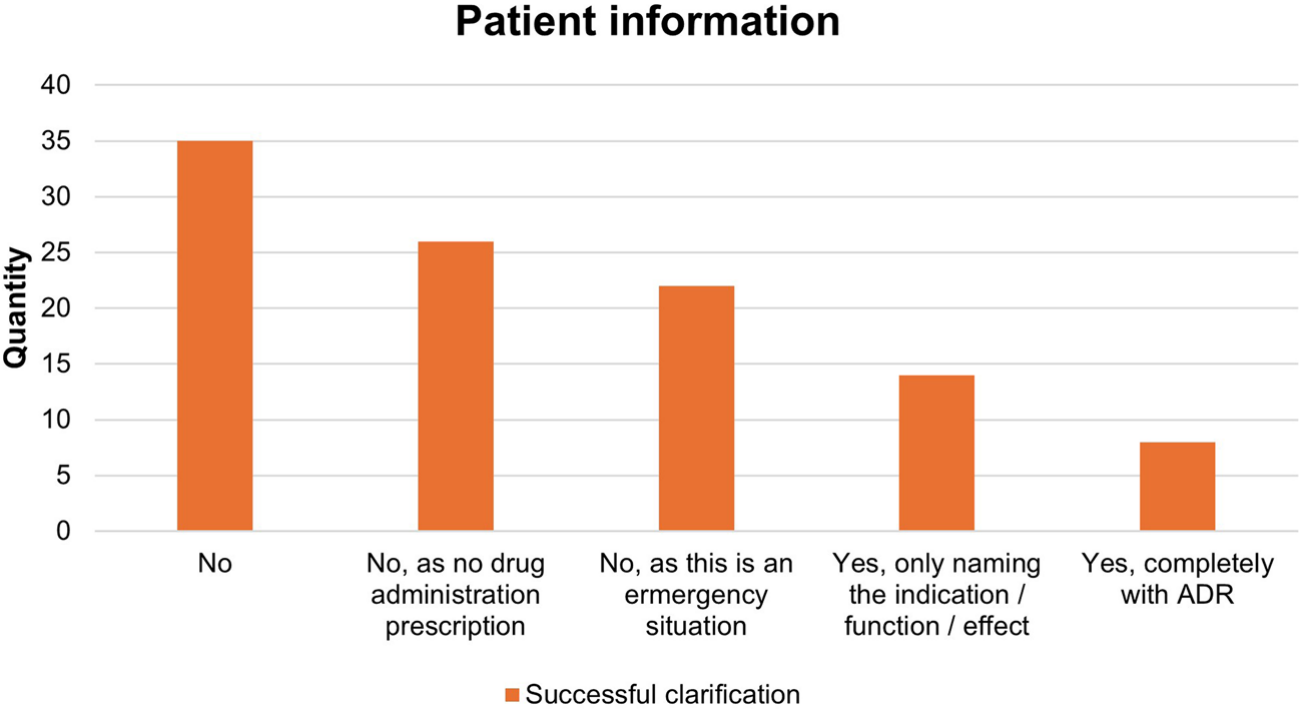
Patient information of the pharmacological therapy

The majority of pharmacological therapies were carried out without informing the patient. Figure 5 shows the number of explanations given. This clearly shows that no information was provided for 35 pharmacological therapies. In 26 items, a therapy was considered but not carried out and therefore no information was required. Similarly, in 22 cases no information was pro, because these were emergency situations. Only in 8 of the 105 pharmacological items complete information were provided to the patient. In particular, the name of the drug and possible adverse drug reactions were mentioned.

### Drug group distribution

**Fig. 6:**
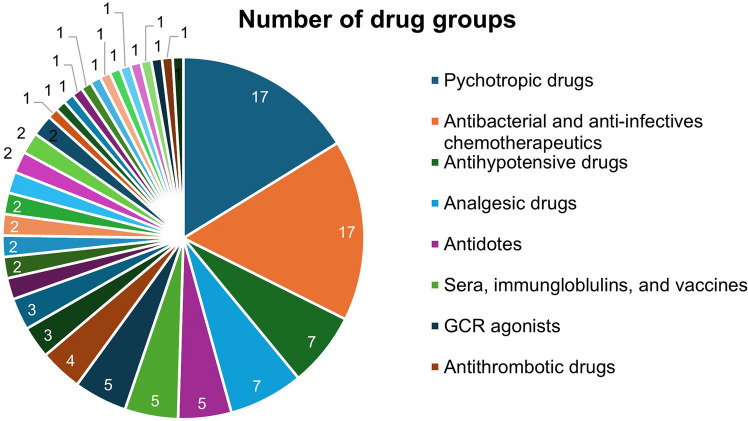
Distribution of the drug groups based on the classification of the red list

Figure 6 shows the respective drug groups covered in the episodes based on the classification of the Red List (Rote Liste 2025) was used here. It shows a predominant use of psychotropic drugs and antibacterial drugs, each with 17 of 105 items.

Prescription figures were used to compare the drugs and drug groups shown with the actual figures. These are based on the Drug Prescription Report (DPR), which was published by the Scientific Institute of the AOK (Wissenschaftliches Institut der AOK (WIdO), 2024). The prescription figures from the respective previous year are presented here. This means that the prescriptions from 2022 are reflected in the DPR 2023. A look at the DPR at the time of broadcast (2004) and the DPR 2023 shows a disproportionate prescription of psychotropic drugs, epinephrine, immunoglobulins and GCR agonists in the television series. Levothyroxine, antithrombotic drugs and COX inhibitors like ibuprofen are underrepresented (Figure S3).

### Presentation of the drug

**Fig. 7:**
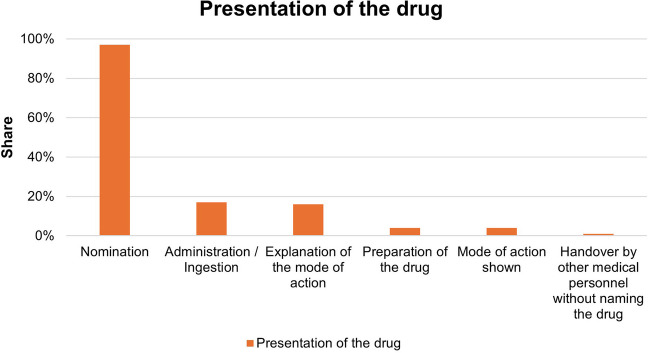
Proportions of the presentation of pharmacological content

The drugs were named in 97% of the episodes analyzed. In 1% of the items, the drug was administered without explicitly naming the drug. In 4% of the pharmacological content, the mode of action of the drug was presented visually. The mode of action was only explained in 16% of cases. Figure 7 shows the proportions of the presentation. Multiple entries are possible.

### Frequency of diagnosis

Each diagnosis appears once in the analyzed episodes. A duplication only occurs in the example of dermal intoxication, where this is caused by two patients with the same intoxication within one episode (Figure S4).

### Drugs as diagnostics

In addition to the therapeutic use of drugs, they are also portrayed as diagnostic drugs in the TV series *Dr. House.* ADRs are accepted as a lesson for developing the diagnostic process leading to the final diagnosis. Three scenes present this approach.

Sudden discontinuation of psychostabilizing medication is also ordered to improve diagnosis, despite knowledge of withdrawal and rebound phenomena. The scenes mentioned are shown in Table 3.

**Table 3:** Presentation of the drugs that were used as diagnostic drugs

Episode	Named drug	Scene presentation
Episode 1, Case 1	high-dose prednisone	Dr. House prescribes high doses of prednisone as he suspects cerebral vasculitis. Unexpected drug effects are accepted by Dr. House as "educational". No explanation is given, only that this is "part of the treatment". When asked several times, the assumption of vasculitis and treatment with GCR agonists (“steroids”) is explained
Episode 4, Case 1	Vancomycin Aztreonam	Dr. House orders vancomycin for MRSA and aztreonam for other bacterial infections, especially Pseudomonas, Haemophilus influenzae and VRE, in two critically ill newborns In the course of the disease, kidney failure occurs without crystal formation, caused by the previously administered antibacterial drugs. As aztreonam and vancomycin can trigger kidney failure, Dr. House decides to discontinue aztreonam in one child and vancomycin in the other to protect the other affected children The aztreonam does not seem to be working, so Dr. House orders a double dose of vancomycin for all affected children
Episode 10, Case 1	Isoniazide ("INH") Rifampicin Streptomycin	Dr. House suspects a tuberculoma in the ovarian mass and orders isoniazide, rifampicin and streptomycin without further diagnostics, as there is no treatment for this patient with the differential diagnosis (advanced ovarian cancer)
Episode 6, Case 1	Psychotropic drugs	Dr. House orders the immediate (sudden) discontinuation of the psychotropic drugs to see what psychological complaints are actually present

### Genre comparison

In 2022 and 2023, the analyses of pharmacological content in the German TV series *Tatort* showed a high level of agreement on the accuracy of pharmacological and toxicological content. The comparison of the names of the drug showed a higher proportion of INN use in the TV series *Dr. House*. Trade names are also used more frequently. In contrast to the *crime scene* and the analysis of crime novels, the use of fictitious names was not observed (figure S5).

Figure S6 provides a selection of examples of the proportions of drug groups classified according to the Red List (Rote Liste 2025). A comparison of the drug groups in the *crime scene* and the actual prescription figures in Germany based on the DPR 2005 (Schwabe and Paffrath 2006) and DPR 2023 (Wissenschaftliches Institut der AOK (WIdO), 2023) shows a disproportionate use of antibacterial drugs in the presentation by *Dr. House.* Psychotropic drugs were also overrepresented, but to a much lesser extent than in the analyses of the *Tatort series*. Narcotic, sedative, hypnotic drugs are also overrepresented in *Tatort* compared to the real prescription figures and *Dr. House.*

In *Dr. House*, drugs are used therapeutically (figure S7). The analysis by Möller and Seifert of crime novels (Möller and Seifert 2024) shows a predominantly toxic use of drugs, similar to the analysis by Ellerbeck and Seifert in *crime scene* (Ellerbeck and Seifert 2022)*.* The systematic analysis of the *crime scene* by Borchert and Seifert (Borchert and Seifert 2023) describes an almost one-third split between therapeutic approaches, intentionally toxic situations and aspects of dependency. In *crime scene* and in the crime novels, there is no use of drugs for diagnostic purposes.

Figure S8 shows the proportion of the dosage form of the respective drug. There is frequent lack of information on this aspect in the TV series *Dr. House*. The studies by Möller and Seifert (Möller and Seifert 2024) and Ellerbeck and Seifert (Ellerbeck and Seifert 2022) show a predominantly oral administration of the drugs. No data are available on the form of administration from the analysis by Borchert and Seifert (Borchert and Seifert 2023).

### Quality of pharmacological content throughout the series

**Fig. 8:**
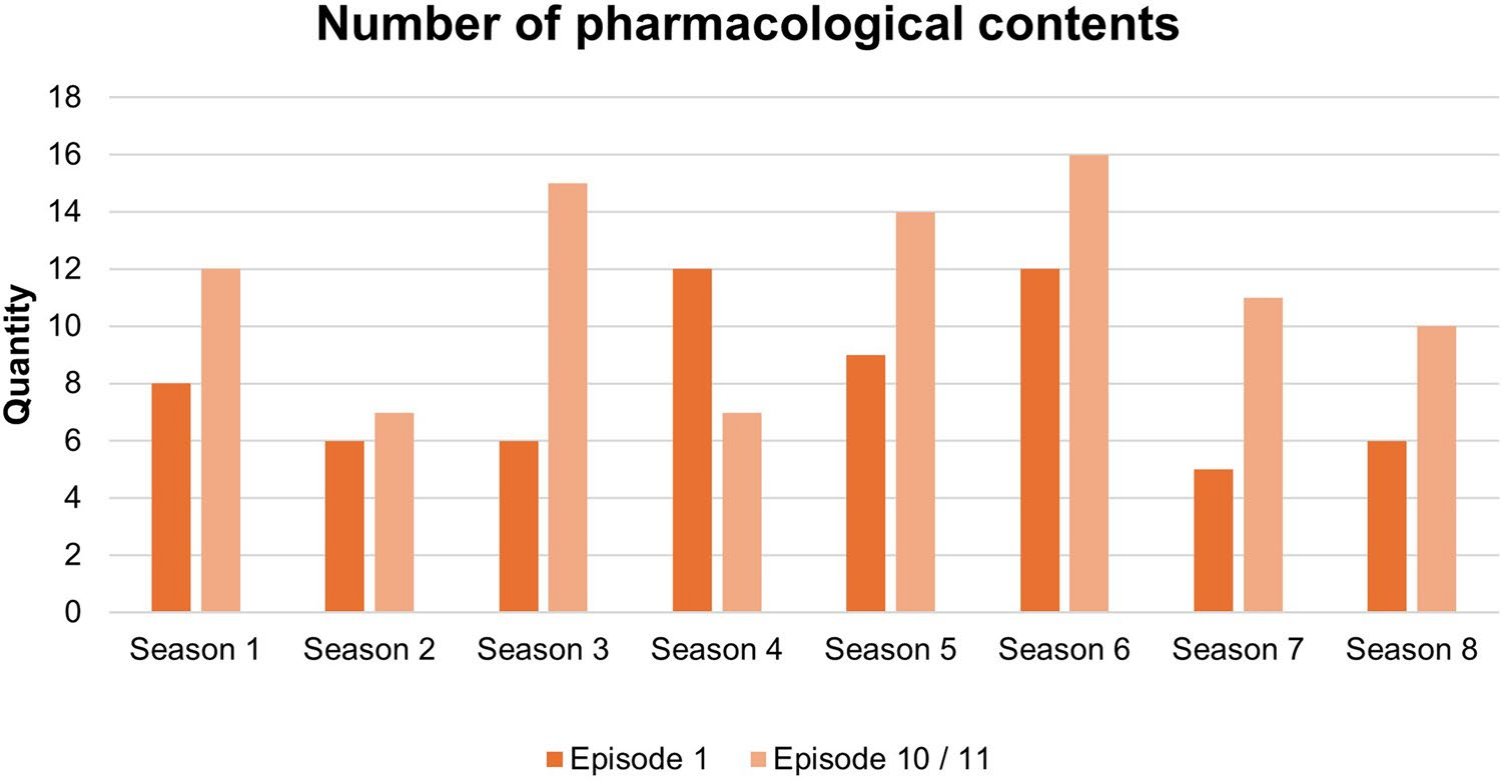
The number of pharmacological content during the series

The number of pharmacological items per episode varies greatly over the course of the series. The number of pharmacological contents ranged between 5 and 16. In the first episode, all seasons except the fourth had fewer items than the eleventh. On average, there are 8.8 analyzed items per episode. This is shown in Fig. 8.

**Fig. 9:**
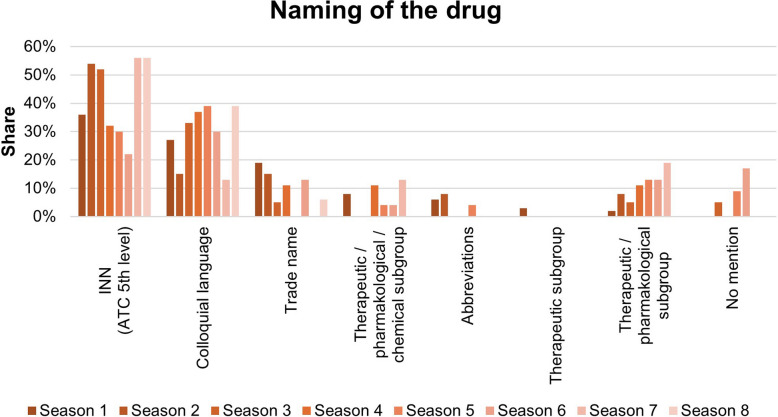
Naming the items according to the ATC code during the series

The naming of drugs is highest in seasons 2, 3, 7, and 8, with over 50% of INN usage. In season 6, INN naming is used in 22% of pharmacological content. Season 6 also shows a 17% non-mention of medication. The use of trade names tended to decline over the course of the series. The use of therapeutic/pharmacological subgroups has risen steadily over the course of the series to 19% in season 7. These terms were not used in season 8. The presentation can be seen in Fig. 9.

**Fig. 10:**
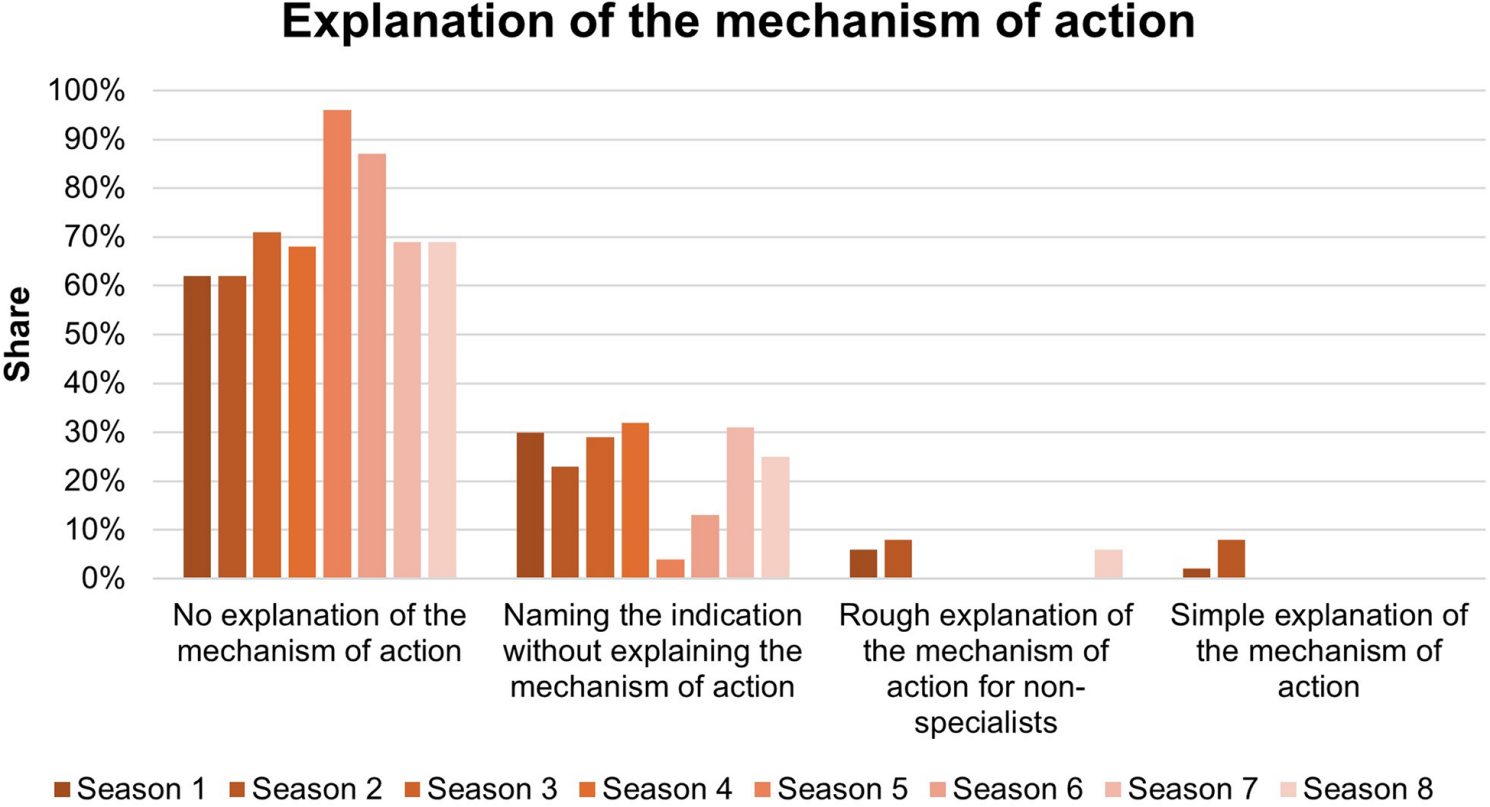
Explanation of the mechanism of action during the series

Figure 10 illustrates the explanation of the mechanism of action throughout the series. Throughout the series, there is largely no explanation of the mechanism of action. Seasons 5 and 6 show rates of 96% and 87% respectively. Only in seasons 1, 2, and 8 is there a small number of explanations of the mechanism of action.

**Fig. 11:**
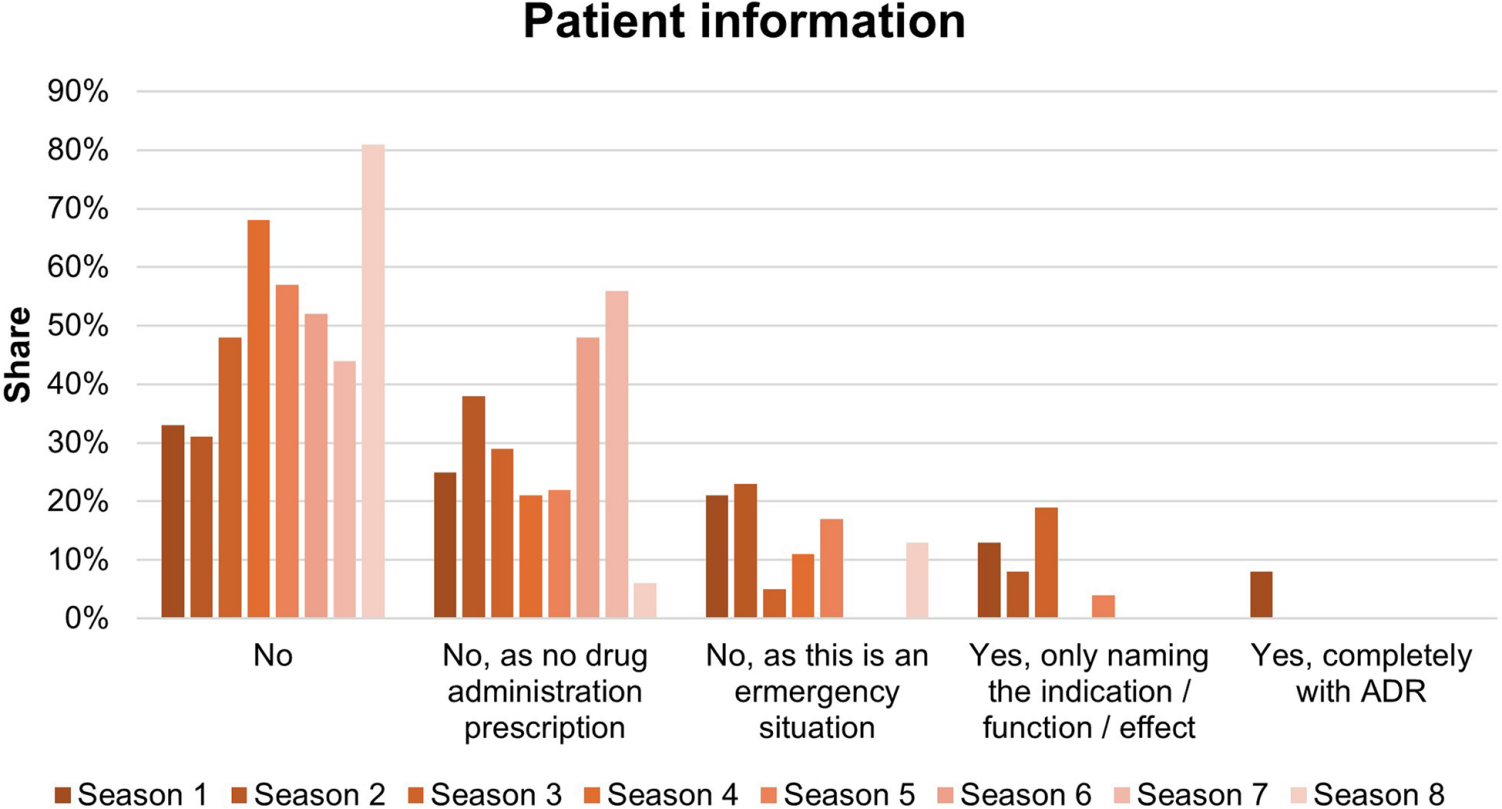
Patient information of the pharmacological therapy during the series

The rate of patient information appears to decrease steadily over the course of the series. The proportion of cases in which no information was provided is 81% in season 8. Patient information with mention of the indication, function, or effect occurred exclusively in seasons 1, 2, 3, and 5. The patient information rates are shown in Fig. 11.

**Fig. 12:**
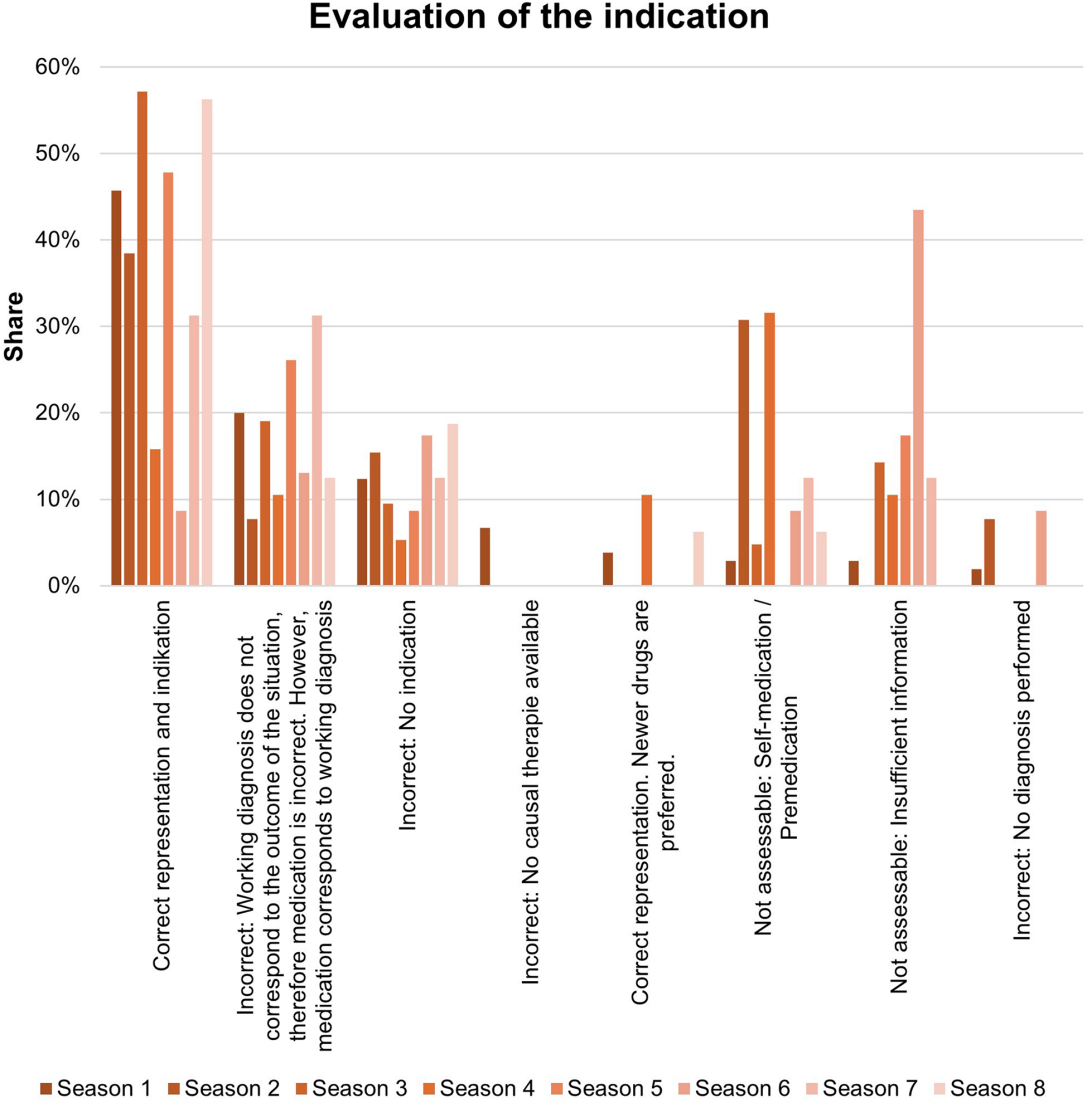
Evaluation of the indication during the series

Figure 12 shows the evaluation of the indication. Seasons 1 and 5 show a rate of correct indication of over 40%. Seasons 3 and 8 show a rate of over 50%. In season 6, a correct indication is shown in 9% of cases. However, in this stage, only 55% of the items could be assessed because there was insufficient information about the active ingredient, pre-existing conditions, or the situation. Seasons 2 and 4 show high rates of self-medication and pre-medication, which could not be assessed due to a lack of background information.

## Conclusions

Overall, the presentation of the pharmacological content in the episodes of the TV series *Dr. House* can be considered reasonably realistic. The drugs are largely named by INN. The uniform language helps to prevent confusion. In comparison to the TV series *Tatort* and the crime novels analyzed, INNs were used more frequently. In contrast to the comparison media mentioned above, there was no use of fictitious names in the TV series *Dr. House*. The indications for the respective working diagnoses are consistent with the literature of the time and the current literature. Particularly noteworthy is the depiction of drugs as diagnostic tools in the television series *Dr. House*. This use of drugs was not depicted in the comparative media. Ethical conflicts arise here. The use of two different drugs in the treatment of two children suffering from a neonatal infection caused by the same pathogen is particularly critical. The idea that at least one of the two therapies could be effective and thus save the life of at least one child is ethically debatable. This scene shows the stubbornness of Dr. House and his priority of solving the diagnostic puzzle. This approach creates tension peaks that are conducive to entertainment.

However, there are also areas in which drug presentation in *Dr. House* is problematic. The use of psychoactive substances in particular shows a major deviation from reality. The use of haloperidol without prior electrocardiographic monitoring to rule out cardiac arrhythmias and subsequent detection of QTc prolongation is contrary to current recommendations and can therefore lead to life-threatening situations. Such a presentation highlights the recklessness of *Dr. House* of accepting ADRs without pointing them out to non-medically educated viewers. Similarly, situations involving the use of benzodiazepines are portrayed in an overly dramatic way.

The presentation of the diagnostic path including the use of drugs to come to a final diagnosis should be emphasized positively. In this way, through trial and error, many drugs are covered in a small sample of episodes.

The impression is that in the first episode of each season, compared to the rest of the season, more story is depicted than medical content. It is particularly striking that in season 6, there is only a low use of INN and a high rate of unnamed drugs. There is also a high rate of non-evaluable content, as there is too little information about the drugs, pre-existing conditions, or indications for pre-medication. In addition, only a small number of drugs were correctly indicated and presented in this season. Seasons 2, 7, and 8 are the most accurate in terms of naming in the analysis. The presentation and explanation of the mechanism of action is only shown to a limited extent in seasons 1, 2, and 8. The rate of information provided steadily decreases over the course of the series. Only season 1 provides information including adverse drug reactions. In summary, the accuracy of the pharmacological content tends to decline, reaching its lowest point in season 6 and improving significantly in seasons 7 and 8.

Thus, pharmacological education for the general audience is accomplished by *Dr. House*. Unfortunately, detailed information on the mechanism of action of drugs and their mode of action is rarely presented. This also holds for missing patient information and mode of drug application. Trained doctors or teaching of students can thus also benefit from the television series in some respects in terms of role modeling and the presentation of (in)appropriate behavior.

## Limitations

By analyzing the first ten episodes of the first season and the first and eleventh episodes of seasons 2 through 8 of *Dr. House*, it is not possible to apply the results to all episodes. In addition, we analyzed the German translation of the series. This could have affected details in content. A further limitation is the incompleteness of the DPR from 2005 (Schwabe and Paffrath 2006), as it was not possible to access all drugs with their corresponding prescription figures.

## Supplementary Information

Below is the link to the electronic supplementary material.

ESM1 (DOCX.162 KB)

## Data Availability

All original data for this study is available from the authors upon reasonable request.

## Abbreviations

ADR: Adverse drug reaction
ALS: Amyotrophic lateral sclerosis
ATC: Anatomical Therapeutic Chemical Classification System
DPR: Drug Prescription Report
INN: International Nonproprietary Name
IvIG: Intravenous immunoglobulin
SSPE: Subacute sclerosing panencephalitis
