# Supplementary material for: Pharmacological content in the television series “House MD”: analysis from a German pharmacologist’s perspective
Source: Naunyn Schmiedebergs Arch Pharmacol. 2026 Feb 7;399(7):10615–41. doi: 10.1007/s00210-026-04995-6 (PMC13152976; doi:10.1007/s00210-026-04995-6)
Supplement: Supplementary file 1 — (DOCX.162 KB) [file 210_2026_4995_MOESM1_ESM.docx]

**Pharmacological content in the television series ‚House MD‘: Analysis from a German pharmacologist's perspective**

**Erika Schmoll and Roland Seifert**

**Abbreviations**

DVT Deep vein thrombosis

SSPE Subacute sclerosing panencephalitis

**Figure S1: Representation of the number of prescriptions issued by Dr. House's team**

**Figure S2: Illustration of the effect on the audience**

**Figure S3: Comparison of selected contents of the prescription figures for drugs and drug groups in the DPR 2005, DPR 2023 and the drugs shown in Dr. House**

**Figure S4: Presentation of diagnoses according to their frequency**

**Figure S5: Selected comparison of the naming of the drug**

**Figure S6: Comparison of the drug groups of different genres**

**Figure S7: Illustration of the intended use of the drugs in comparison with the crime scene and the crime novels analyzed**

**Figure S8: Comparison of the forms of presentation of the different genres**

**Table S1: Tabular summary of the assessments of Dr. House by professionals**

| **Assessment** | **Author** |
| --- | --- |
| *"Dr. House* is technically brilliant and a **gifted diagnostician**, **but** his human and **communication skills are fatally underdeveloped**"  "With House, we can show future doctors how not to do it."  (Spiegel.de, 2010) | Harald Hayner, research assistant Institute for Ethics and  Communication in Healthcare - Private University Witten-Herdecke |
| "The **first two seasons were brilliant**, but now the series is visibly losing its level."  (Focus.de, 2013) | Dr. Jürgen Schäfer  Academic Director of the Philipps University of Marburg  Cardiologist and endocrinologist |
| "When it's either entertainment or medical accuracy, entertainment always wins"  (Focus.de, 2013)  "**There are many unrealistic aspects** to the show. For example, neither nurses nor technicians work in the series hospital. The doctors order tests themselves, carry them out themselves and interpret them afterwards. They even carry out routine tasks such as taking blood samples. But the craziest thing is that they break into patients' homes to find out something about them. On the other hand, the series is very realistic where it counts the most, namely in **finding a diagnosis**."  (Focus.de, 2013) | Lisa Sanders  Medical advisor for production |
| "In his **uncompromising** and perceptive manner, Dr. Gregory House gets to the heart of some - albeit completely **unethical** - **truths of everyday hospital life** that many doctors don't even dare to think about, let alone say, but sometimes would like to."  (Tuffs, 2006) | Annette Tuffs  Television review:  *Dr. House* - Misanthrope with cult status |
| "Of the current medical shows on TV, **House** is **probably the most accurate**. It's definitely way above average for the genre."  (Focus.de, 2013) | Scott Morrison  Physician from Illinois  The blog created by S. Morrison for this purpose can no longer be viewed.  (Morrison, no date) |
| "Good entertainment **can save lives."**  "There are diagnoses that are extremely consistent."  "Some of the cases are so well researched that I sometimes have to look them up myself."  (Spiegel.de, 2014)  (Focus.de, 2015) | Dr. Jürgen Schäfer  Academic Director of the Philipps University of Marburg  Cardiologist and endocrinologist |

**Table S2: Episodes included in the analysis, including titles, diagnoses and first broadcast dates**

| **Episode** | **Title US / Germany** | **Diagnoses** | **First broadcast USA** | **First broadcast in Germany** |
| --- | --- | --- | --- | --- |
| 1 | Pilot / Schmerzensgrenzen | Neurocysticercosis,  back pain, asthma,  fatigue syndrome | 16.11.2004 | 01.05.2006 |
| 2 | Paternity /  Falsche Geschichte | SSPE,  wound infection | 23.11.2004 | 08.05.2006 |
| 3 | Occams Razor /  Das Ende danach? | Colchicine poisoning | 30.11.2004 | 15.05.2006 |
| 4 | Maternity /  Nichts hilft | Viral infection,  Unplanned pregnancy | 07.12.2004 | 22.05.2006 |
| 5 | Damned If You Do /  Nur die Braut Christi? | Allergy,  Diarrhea | 14.12.2004 | 29.05.2006 |
| 6 | The Socratic Method /  Schizophren? | TVT,  Wilson's disease | 21.12.2004 | 05.06.2006 |
| 7 | Fidelity /  Fremd- und nicht gut gegangen | African trypanosomiasis, intoxication by β-AR- antagonists | 28.12.2004 | 20.06.2006 |
| 8 | Poison /  Geiz ist Gift | Phosdrin intoxication, neurosyphilis | 25.01.2005 | 27.06.2006 |
| 9 | DNR /  Leben wider Willen | Arteriovenous malformation, diabetes mellitus type II | 01.02.2005 | 04.07.2006 |
| 10 | Histories /  Letzte Suche | Rabies,  Korsakov syndrome | 08.02.2005 | 11.07.2006 |

**Table S3: ATC classification of the Scientific Institute of the AOK (WIdO) in cooperation with the WHO.**

| **ATC code level** | **Meaning** | **Example code** | **Example** |
| --- | --- | --- | --- |
| 1st level | main anatomical group | A | Alimentary system and metabolism |
| 2nd level | therapeutic subgroup | A10 | Antidiabetics |
| 3rd level | therapeutic / pharmacological subgroup | A10B | Antidiabetics, excl. insulins |
| 4th level | chemical / therapeutic / pharmacological subgroup | A10B B | Sulfonylureas |
| 5th level | subgroup for chemical substances | A10B B01 | Glibenclamide |

**Table S4: Comparison of the accuracy of the indications in current literature and the information in specialist literature at the time of broadcast. For this purpose, “Brunton, L. L., Lazo, J. S., & Parker, K. L. (2006). Goodman & Gilman's The Pharmacological Basis of Therapeutics (11th ed.). McGraw Hill” was used.**

| **Category** | **Current source** | **Goodman & Gilmans´*** |
| --- | --- | --- |
| Correct representation and indication | 48 | 48 |
| Correct representation. Newer drugs are preferred in the above-mentioned textbook. | 4 | 1 |
| Not assessable: Self-medication/premedication. | 3 | 3 |
| Not assessable: Insufficient information | 3 | 6 |
| Incorrect: Working diagnosis does not correspond to the outcome of the situation, therefore medication is incorrect. However, medication corresponds to working diagnosis. | 21 | 21 |
| Incorrect: No causal therapy available | 7 | 7 |
| Incorrect: No diagnosis is made | 2 | 2 |
| Incorrect: No indication | 14 | 14 |
| Incorrect: Self-medication/premedication without indication | 3 | 3 |
| **Result** | **105** | **105** |
